# A novel model to study mechanisms of cholestasis in human cholangiocytes reveals a role for the SIPR2 pathway

**DOI:** 10.1097/HC9.0000000000000389

**Published:** 2024-02-26

**Authors:** Diana Islam, Izza Israr, Mohamed A. B. Taleb, Aditya Rao, Robel Yosief, Rukhsar Sultana, Fotios Sampaziotis, Olivia C. Tysoe, Michael Trauner, Saul J. Karpen, Anand Ghanekar, Binita M. Kamath

**Affiliations:** 1 Development & Stem Cell Biology program, Peter Gilligan Centre for Research and Learning, The Hospital for Sick Children, Toronto, Ontario, Canada; 2 Wellcome–MRC Cambridge Stem Cell Institute, Department of Medicine, University of Cambridge, Cambridge, Cambridgeshire, UK; 3 Department of Medicine, University of Cambridge, Cambridge, Cambridgeshire, UK; 4 Hans Popper Laboratory of Molecular Hepatology, Department of Internal Medicine III, Division of Gastroenterology and Hepatology, Medical University of Vienna, Vienna, Austria; 5 Division of Pediatric Gastroenterology, Department of Pediatrics, Hepatology, and Nutrition, Children’s Healthcare of Atlanta and Emory University School of Medicine, Atlanta, Georgia, USA; 6 Division of General Surgery, Department of Surgery, University Health Network & The Hospital for Sick Children, University of Toronto, Toronto, Ontario, Canada; 7 Division of Gastroenterology, Hepatology and Nutrition, Department of Pediatrics, The Hospital for Sick Children and the University of Toronto, Toronto, Canada

## Abstract

**Background:**

Ductular reactivity is central to the pathophysiology of cholangiopathies. Mechanisms underlying the reactive phenotype activation by exogenous inflammatory mediators and bile acids are poorly understood.

**Methods:**

Using human extrahepatic cholangiocyte organoids (ECOs) we developed an injury model emulating the cholestatic microenvironment with exposure to inflammatory mediators and various pathogenic bile acids. Moreover, we explored roles for the bile acid activated Sphingosine-1-phosphate receptor 2 (S1PR2) and potential beneficial effects of therapeutic bile acids UDCA and norUDCA.

**Results:**

Synergistic exposure to bile acids (taurocholic acid, glycocholic acid, glycochenodeoxycholic acid) and TNF-α for 24 hours induced a reactive state as measured by ECO diameter, proliferation, lactate dehydrogenase activity and reactive phenotype markers. While NorUDCA and UDCA treatments given 8 hours after injury induction both suppressed reactive phenotype activation and most injury parameters, proliferation was improved by NorUDCA only. Extrahepatic cholangiocyte organoid stimulation with S1PR2 agonist sphingosine-1-phosphate reproduced the cholangiocyte reactive state and upregulated S1PR2 downstream mediators; these effects were suppressed by S1PR2 antagonist JET-013 (JET), downstream mediator extracellular signal-regulated kinase 1/2 inhibitor, and by norUDCA or UDCA treatments. JET also partially suppressed reactive phenotype after bile acid injury.

**Conclusions:**

We developed a novel model to study the reactive cholangiocyte state in response to pathological stimuli in cholestasis and demonstrated a contributory role of S1PR2 signaling in both injury and NorUDCA/UDCA treatments. This model is a valuable tool to further explore the pathophysiology of human cholangiopathies.

## INTRODUCTION

Cholangiopathies are a group of liver diseases that share cholangiocytes as their primary target and have no highly effective or curative treatments. Therapies in use for cholangiopathies include several bile acid treatments. Chief among bile acid–based therapies in use for decades is ursodeoxycholic acid (UDCA), a hydrophilic bile acid present in limited amounts in human bile with both choleretic and anti-inflammatory properties.^1^ More recently, norursodeoxycholic acid (norUDCA, recently renamed as norucholic acid), a side chain shortened bile acid derivative with potent choleretic and anti-inflammatory activity in mouse models of cholestasis, is being tested in several forms of cholestatic diseases.^2^,^3^,^4^,^5^,^6^ Since norUDCA is resistant to side chain conjugation, it does not require conjugated bile acid transporters to enter the bile acid pool and appears to engage the cholehepatic shunt.^7^

The pathophysiological basis of cholangiopathies is complex. After injury, cholangiocytes develop a common general reactive phenotype involving the secretion of a variety of cytokines, chemokines, growth factors, and other mediators.^8^,^9^,^10^ Once stimulated, these cholangiocyte-derived secreted factors recruit a broad array of resident and circulating responding cells including lymphocytes, stellate cells, peribiliary fibroblasts, and macrophages, thereby promoting inflammation, biliary damage, and fibrosis.^11^,^12^,^13^,^14^ Subsequently, the reactive ductular phenotype further enhances cholangiocyte damage by bidirectional communication of mediators with newly recruited inflammatory cells.

Cholangiocytes are exposed to high concentrations of bile acids in normal physiology, principally on the apical surface facing the biliary compartment. In cholestatic states, the intrahepatic concentrations of bile acids rise, and the composition shifts to more hydrophobic and toxic bile acid species with physical exposure of basolateral as well as apical surfaces of cholangiocytes to bile acids, other biliary solutes, and various responsive mediators in the cholestatic liver from adjacent peribiliary cells and hepatocytes.^15^ This combinatorial exposure of cholangiocytes to multiple surrounding molecules is a common pathogenic feature of many cholangiopathies.^16^,^17^,^18^ In addition to exposure to high concentrations of bile acids during cholestasis, cholangiocytes are also exposed to increased concentrations of proinflammatory cytokines that mediate reactive responses. Cell culture and animal studies indicate that cholangiocytes may be more susceptible to combined stimulation of bile acids and inflammatory mediators than to either of these alone.^19^,^20^ While bile acids mediate their injurious effects through a variety of mechanisms,^21^,^22^,^23^ recent studies have revealed that the transmembrane sphingosine-1-phosphate receptor 2 (S1PR2) may play a novel and central role in mediating bile acid and inflammation-mediated cholestatic pathology in vivo. S1PR2 responds to select conjugated bile acids to elicit specific patterns of signal transduction (including extracellular signal-regulated kinase [ERK] and serine/threonine kinase (AKT) pathways) in cholangiocytes.^24^,^25^ In addition, some recent studies have reported upregulation of S1PR2 mRNA expression in liver samples from patients with biliary atresia.^26^,^27^

Since the reactive cholangiocyte phenotype has been predominantly studied in animal models, the effects of bile acids on human cholangiocyte injury and activation of their reactive phenotype are poorly understood. In addition, the relevance of animal studies is limited because animals have bile acid compositions that are significantly different from humans^28^ and animal studies have shown conflicting results.^29^,^30^

The aim of this study was to develop an in vitro primary human cholangiocyte model that mimics key features of the cholestatic microenvironment. We used well-characterized extrahepatic bile duct–derived primary human cholangiocyte organoids that retain the morphologic, phenotypic, and functional characteristics of cholangiocytes over multiple passages.^31^ These cells were exposed to elevated human bile acid concentrations together with increased proinflammatory cytokine levels in order to mimic key components of cholangiopathies to study cholangiocyte injury and activation of the reactive cholangiocyte phenotype. We also investigated the role of the S1PR2 pathway in cholangiocyte injury, reactive phenotype activation, and responses to bile acid–based therapies UDCA and norUDCA as a means to determine if these 2 therapies can elicit effective anti-cholestatic features in a controlled human cholangiocyte model.

## METHODS

### Human extrahepatic cholangiocyte organoid culture, injury, and treatments

Human extrahepatic cholangiocyte organoids (ECOs) were generated from cholangiocytes isolated from the normal extrahepatic bile ducts of deceased organ donors as described before, tissue samples were excised after obtaining informed consent from the donor’s family in accordance with research ethics committee approval.^31^ ECOs were propagated according to the described protocol^31^ in serum-free William E medium under 5% CO_2_ and ambient O_2_; passaged every 5–6 days by dissociation in cell recovery solution (Corning) for 30 minutes at 4°C, then collected by centrifugation at 300 g for 3 minutes, washed, and replated to form organoids. Two ECO lines were used for the experiments, data shown are combined results. ECO lines were amplified and frozen between passages 6 and 8. For each experiment, frozen cells were thawed and passaged at least 3 times. All experiments were performed within 15 passages and after organoids were allowed to grow for 5 days.

The human liver has concentrations of taurocholic acid (TCA), glycocholic acid (GCA), and glycochenodeoxycholic acid (GCDCA) ranging from 1 to 4 mM.^18^ We first optimized injurious bile acid concentrations by exposing ECOs to either vehicle (DMSO) or individual bile acids (TCA, GCA, GCDCA; Sigma-Aldrich) ranging from 0.5 to 5 mM for 24 hours in William E medium. Using the information from these pilot experiments, ECOs were exposed to vehicle (DMSO for bile acids, PBS for TNF-α) or the optimal target doses of TCA (1 mM) or GCA (1 mM) or GCDCA (0.5 mM) individually; with or without costimulation of 20 ng/mL TNF-α (Peprotech) for 24 hours. NorUDCA and UDCA’s^2^ effective dosages for the TNF-α+TCA injury model were determined by dose-finding studies. To study potential recovery from the TNF-α+TCA injury model, either vehicle (DMSO) or 250 µM norUDCA or 62.5 µM UDCA were given 8 hours after injury induction by replacement of 50% of the media with fresh media containing the respective treatments for 24 hours. Experiments were performed in duplicate and repeated from 6 to 12 times.

To explore the S1PR2 pathway, ECOs were exposed to (1) vehicle (DMSO); (2) S1PR2 agonist 10 µM sphingosine-1-phosphate (S1P, 6270, Cayman Chemical); (3) 1 mM TCA; (4) 20 ng/mL TNF-α; or (5) 1 mM TCA + 20 ng/mL TNF-α in the presence or absence of the S1PR2 antagonist 10 µM JTE-013 (sphingosine 1-phosphate receptor 2/4 antagonist [JTE}, 10009458, Cayman Chemical) for 24 hours. In parallel sets, ECOs were exposed to either vehicle or 10 µM S1P with or without ERK inhibitor 2 µM U0126 (109511582, Tocris) for 24 hours. For rescue experiments, after either vehicle or 10 µM S1P exposure for 8 hours, ECOs were treated by 50% media replacement as noted with either vehicle or 250 µM norUDCA or 62.5 µM UDCA for 24 hours. Experiments were performed in duplicate and repeated from 4 to 8 times.

### Cyst diameter measurement

ECOs were imaged at the beginning (T0) and end (T24) of treatments using a brightfield, inverted digital microscope (Thermo-Fisher) at ×40 magnification. Image analysis was performed in Image J2 software.^32^ Diameters (as a morphological indicator of injury) were measured for 20–40 ECOs/sample. For each ECO, diameter change was calculated as diameter at T24–T0. The average diameter change was calculated per sample and then per group. Fold change in diameter was calculated as (average diameter change in treatment)/(average diameter change in vehicle control).

### Cell proliferation

ECOs were cultured in glass bottom chamber slides (80807, IBIDI). Ki67, a well-known nuclear marker for cells undergoing mitosis^33^ was used to detect proliferating cells. An immunostaining assay was conducted as described^31^ using an anti-Ki67 antibody (NB500-170, NovusBio) with Alexa 488 (Invitrogen) secondary antibody. DAPI (Invitrogen) was used to label nuclei. Images were acquired with a spinning disc confocal microscope (Leica) at ×200 magnification and analyzed in Velocity 7 software (Quorum Technologies Inc.). The percentage of Ki67-positive cells in each image field was calculated as ([no. of Ki67-positive nuclei]/[no. of DAPI-positive nuclei] × 100). Six to eight random fields/samples were counted for analysis.

### Organoid permeability assay

Organoids grown on glass bottom chamber coverslips (ibidi) were used for live imaging with Leica SP8 lightning confocal microscope (Leica, Wetzlar, Germany) inside humidified 37°C chambers with 5% CO_2_. The imaging protocol was established based on the previously published method.^34^ Briefly, before image acquisition, 1 mM lucifer yellow dye (L0144, Sigma Aldrich) was added to the culture media. Images were acquired in 10 random fields with organoids for 20 minutes at 5-minute intervals after dye addition, at ×200 magnification. Image analysis was performed using Leica LAS X core software. Average background baseline fluorescence was measured from images acquired 1 minute after dye addition. For quantification, the background-subtracted mean fluorescence intensities at 20 minutes were measured inside and outside the organoids within a set region of interest. % Dye intensity inside organoids was calculated by dividing fluorescence intensity inside with total intensity inside + outside organoids. Ten random organoid fields were imaged/sampled in 6 experiments.

### Gene expression

Total RNA was extracted in TRIzol (Invitrogen). PuroSPIN total RNA purification kit (NK051-50, Luna Nanotech) was used for RNA purification and genomic DNA removal. Superscript IV first-strand synthesis system (Invitrogen) was used for reverse transcription. Gene expression was analyzed by quantitative real-time PCR using quantitative real-time PCR kit (800-435-UL, Wisent). Primers are listed in Supplemental Table S1, http://links.lww.com/HC9/A813. Gene expression is calculated using the 2^−ΔΔCT^ method^35^ by normalizing against the vehicle control group as 1-fold and relative to the housekeeping gene glyceraldehyde-3-phosphate dehydrogenase.

### Cytotoxicity

Lactate dehydrogenase (LDH) release is a hallmark of cell death. An LDH-Cytox Assay Kit (426401, BioLegend) was used to access cytotoxicity by measuring LDH activity in cell culture media following 24-hour treatment. The assay was performed according to the manufacturer’s instructions; total cell lysate and cell culture media from vehicle control groups were used as high and low controls, respectively.

### ERK1/2 phosphorylation

The kinase activity of ERK proteins is regulated by dual phosphorylation at Threonine 202 and Tyrosine 204 in ERK1, and Threonine 185 and Tyrosine 187 in ERK2. ERK1 and 2 phosphorylation at these sites and total endogenous ERK1/2 levels were measured independently by ELISA Kit (ab176660, ABCAM).^36^ Cell lysate was collected 24 hours after treatments and assayed according to the manufacturer’s instructions. The percentage of phosphorylated ERK1/2 was expressed relative to total ERK1/2.

### Statistical analysis

Experiments were repeated at least 4 times and the data were reported as mean±SD. One-way ANOVA was used to analyze the variation between groups followed by multiple comparisons test by either Dunnet, Benjamini, Fisher’s least significant difference, or student *t* test, where appropriate, using GraphPad Prism (Graph-Pad, San Diego, CA). A value of *p*<0.05 was considered statistically significant.

## RESULTS

### Bile acids and TNF-α exposure induce cholangiocyte injury and the reactive phenotype

In order to determine the response of ECOs to bile acids, we first performed dose-finding cytotoxicity studies for TCA, GCA, and GCDCA. As shown in Supplemental Figure S1, http://links.lww.com/HC9/A813, the concentrations of 1 mM TCA, 1 mM GCA, and 0.5 mM GCDCA were chosen since these were determined to be optimal for injury induction while minimizing cell death (<5% increase in cell death and <10% increase in LDH activity compared to vehicle control; *p*<0.05). In pilot studies, we exposed ECOs up to 72 hours with lower 100 and 200 µM doses of TCA without detecting significant changes in injury parameters (data not shown).

Exposing ECOs to these 3 bile acids for 24 hours led to alterations in ECO cyst diameter, proliferation, senescence, and membrane permeability. When ECOs were exposed to 1 mM TCA, 1 mM GCA, or 0.5 mM GCDCA, cyst diameters were reduced by 2.9±0.7-, 3.8±0.6-, and 5.5±0.6-fold, respectively, compared to vehicle. When these same 3 bile acids were individually coincubated with 20 ng/mL TNF-α, cyst diameters decreased 3.5±0.6- (TCA), 4.8±0.6- (GCA), and 5.4±0.6-fold (GCDCA), respectively (*p*<0.05) (Figure 1A). Compared to vehicle, Ki67-positive cells (a marker of the proliferative state) decreased by 18.7±3.8, 25.5±3.8, and 21±13.8% after TCA, GCA, or GCDCA exposure, respectively. Combined exposure of TNF-α and TCA, GCA, or GCDCA reduced proliferation more so than bile acids alone, 29±3.8, 27.4±3.8%, and 30.2±3.8%, respectively, compared to vehicle (Figure 1B). We also observed that bile acid exposure upregulated mRNA expression of the cellular senescence markers p16 and p21.^37^ TCA, GCA, and GCDCA exposure increased p16 gene expression by 1.41±0.59-, 1.43±0.44-, and 4.59±1-fold, respectively; and p21 expression by 0.92±0.2-, 0.45±0.2-, and 3.5±1.05-fold respectively compared to vehicle. TNF-α costimulation with each of these bile acids produced further upregulation in p16 expression compared to either TNF-α or bile acid stimulation alone (Figure 1C), suggesting synergistic effects of the combined injury in cellular senescence.

Figure 1Primary human cholangiocyte injury model. Extrahepatic cholangiocyte organoids were exposed to either Veh control or different concentrations of bile acids (1 mM TCA or 1 mM GCA or 500 µM GCDCA) with or without 20 ng/mL TNF-α for 24 hours. (A) Brightfield images of extrahepatic cholangiocyte organoids taken 24 hours after exposure depicting morphologic changes. Cyst diameter measured before and after exposure shows a decline in cyst size after injury, graphed relative to Veh, 20–40 extrahepatic cholangiocyte organoids measured/sample. (B) Immunostaining with cell proliferation marker Ki67 (green) and nuclear marker DAPI (blue) indicating a decline in cell proliferation after injury. Proliferation is quantified as % Ki67+ cells relative to total cells/field, 6–8 random fields/samples were counted. (C) Gene expression analysis by quantitative real-time PCR demonstrating upregulation of senescence markers p16 and p21 after bile acid and TNF-α exposure, graphed as fold change relative to Veh control. (D) Organoid permeability measured by membrane-impermeable fluorescent dye Lucifer yellow internalization assay shows increased permeability after injury. Twenty minutes after adding 1 mM Lucifer yellow to the culture media, dye intensity inside the organoids was measured relative to total fluorescence in the region of interest. The graph depicts an average of 10 random fields/samples. Arrows indicate regions with dye internalization. (E) Gene expression analysis by quantitative real-time PCR demonstrating upregulation of several known reactive phenotype markers after bile acid and TNF-α exposure, graphed as fold change relative to Veh control. (F) % LDH activity in cell culture media indicating increased cytotoxicity after injury, quantified using LDH assay. N = 4–8, error bars represent SD, *p*<0.05, * compared to Veh; # compared to Veh and TNF-α alone; ϕ compared to Veh and both TNF-α and bile acid alone. Abbreviations: BF, bright field; GCA, glycocholic acid; GCDCA, glycochenodeoxycholic acid; MCP-1, monocyte chemoattractant protein-1; TCA, taurocholic acid; Veh, vehicle.

**Figure:**
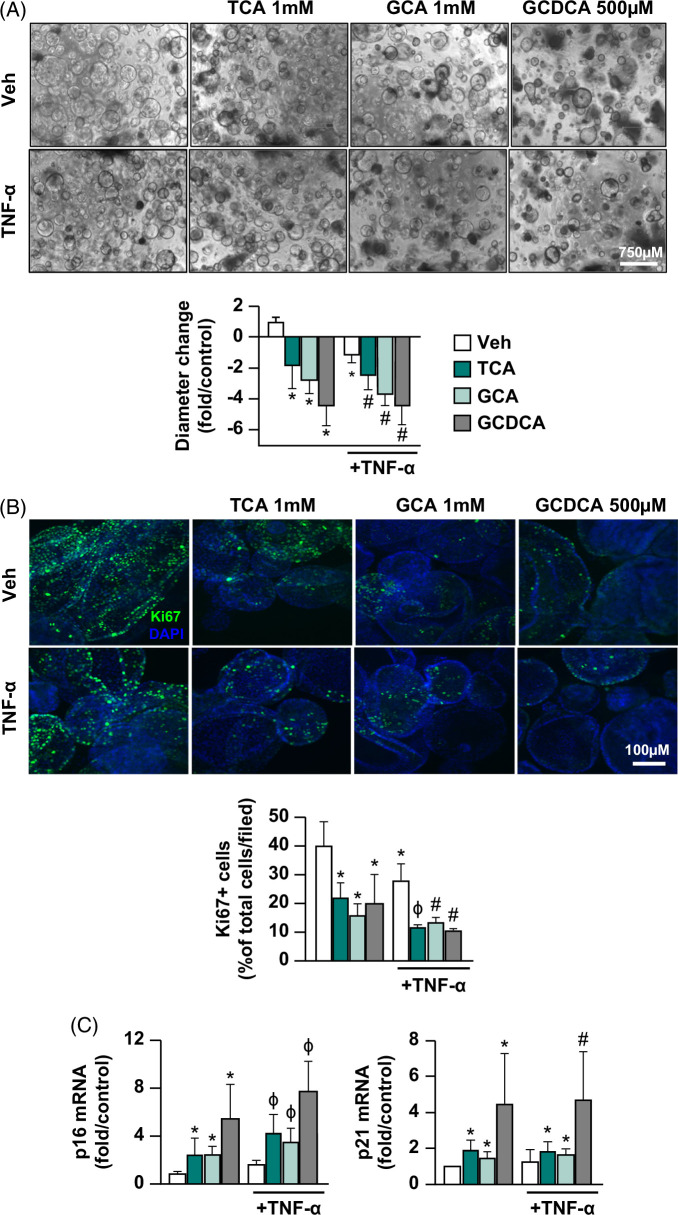

**Figure:**
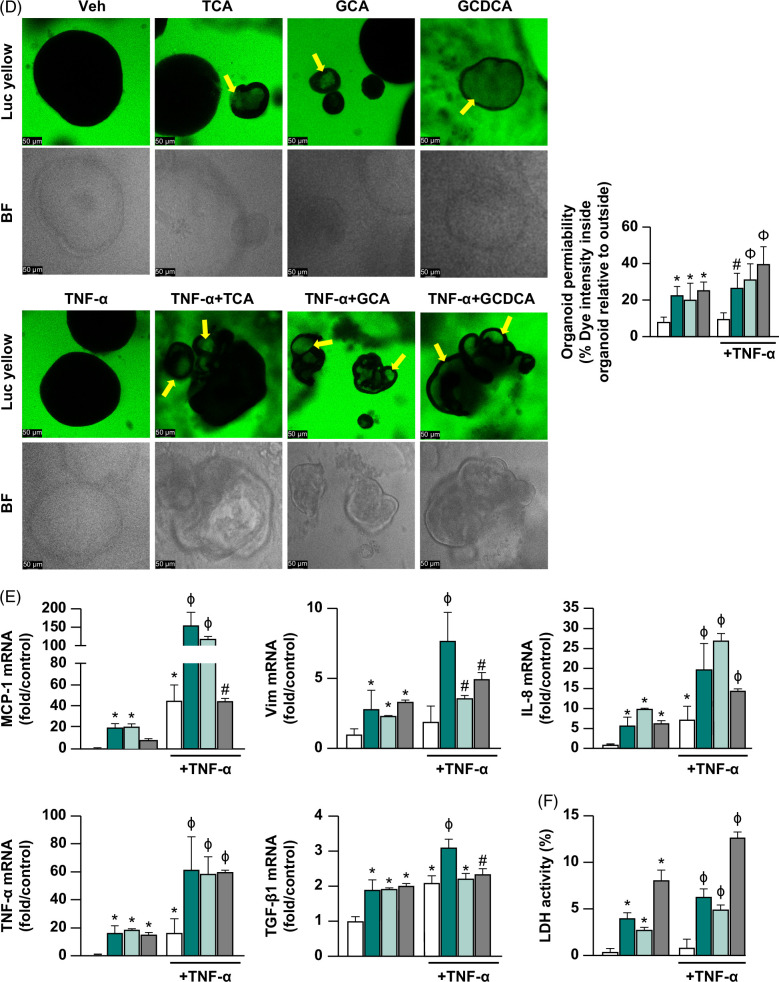

To evaluate the potential impact of cell death in our model, we measured caspase-3 expression and activity. Immunostaining assay showed between 1% and 3% cleaved caspase-3–positive cells after GCDCA stimulation, and even lower expression after TCA and GCA stimulation (Supplemental Figure S2A, B, http://links.lww.com/HC9/A813). There were also no significant changes in caspase-3 activity (Supplemental Figure S2C, http://links.lww.com/HC9/A813). Taken together, these data suggest a minimal contribution of apoptotic cell death in our injury model.

An organoid permeability assay was used to measure membrane-impermeable fluorescent dye lucifer yellow internalization after injury induction, with measurements taken 20 minutes after dye addition. Organoids exposed to vehicle or TNF-α had undetectable amounts of dye inside while TCA, GCA, and GCDCA exposure increased the % dye intensity inside the organoids by 14.5±3.55, 12.17±3.5, and 17.36±3.5%, respectively, compared to vehicle, suggesting bile acid–induced increase in permeability. TNF-α costimulation further increased dye internalization compared to bile acid alone in most groups (Figure 1D). These data suggest some synergistic effects of combined injury in increasing organoid permeability.

Gene expression analysis of known cholangiocyte reactive phenotype markers was employed to explore whether these treatments led to a reactive phenotype. As test genes, we focused upon mRNA levels of chemokines monocyte chemoattractant protein-1 (MCP-1) and IL-8, cytokines TNF-α and TGF-β1, and vimentin (Vim). Compared to vehicle, TCA, GCA, or GCDCA exposure upregulated mRNA expression of all tested markers (*p*<0.05). Compared to either bile acids or TNF-α alone, TNF-α+TCA upregulated expression of all the reactive phenotype markers; TNF-α+GCA upregulated MCP-1, IL-8, and TNF-α expressions, while TNF-α+GCDCA upregulated IL-8 and TNF-α expressions (*p*<0.05) (Figure 1E). Compared to vehicle, LDH activity increased by 3.6±0.4, 2.4±0.5, and 7.7±0.5%, respectively, after TCA, GCA, or GCDCA exposure; and combination with TNF-α increased LDH activity by 5.9±0.4, 4.6±0.5, and 12.3±0.5%, respectively (*p*<0.05) (Figure 1F). Taken together, these findings indicate that individual hydrophobic bile acid treatment of ECOs reduced organoid cyst diameter, reduced cell proliferation, increased organoid permeability, increased cellular senescence, LDH activity, and increased sentinel mRNA expression underlying the reactive phenotype. Combined exposure of bile acids with TNF-α led to synergistic effects by further increasing many of these injury parameters like senescence gene p16 expression, LDH activity, and expression of select reactive phenotype markers, especially IL-8 and TNF-α.

### Protective effects of norUDCA and UDCA treatment in the primary human cholangiocyte injury model

We first explored optimal effective and potentially toxic doses of both norUDCA (250–1000 µM) and UDCA (62.5–250 µM) in the standard TNF-α+TCA injury model. As seen in Supplemental Figure S3, http://links.lww.com/HC9/A813, 250 µM norUDCA and 62.5 µM UDCA elicited effective suppression of IL-8 while leading to low levels of LDH activity. Therefore, these doses were used as treatments of ECOs for subsequent experiments.

We examined the effects of norUDCA and UDCA treatments on cyst diameter, cell proliferation, reactive phenotype marker gene expressions, and LDH activity. NorUDCA or UDCA treatments given after vehicle stimulation had no significant effects on cyst diameter (Figure 2A), cellular proliferation (Figure 2B), reactive phenotype markers (Figure 2C), or LDH activity (Figure 2D) compared to vehicle alone. Compared to TNF-α+TCA, treatment with norUDCA+TNF-α+TCA improved cyst diameter 5.9±1.4-fold (Figure 2A), improved cell proliferation (Ki67-positive cells) 8.8±3.6% (Figure 2B), suppressed gene expression of the reactive phenotype markers MCP1, Vim, IL-8, TNF-α, and TGF-β1 (47±3.3-, 6.5±1-, 3.4±0.8-, 22±5.4-, and 0.33±0.07-fold, respectively) (Figure 2C), and reduced LDH activity 5.5±0.5% (Figure 2D) (*p*<0.05). Compared to TNF-α+TCA, UDCA+TNF-α+TCA treatment improved cyst diameter 4.3±1.4-fold (Figure 2A), suppressed expression of the reactive phenotype markers MCP-1, Vim, IL-8, TNF-α, and TGF-β1 (48.7±3.7-, 6.1±1.1-, 3.5±0.9-, 20.1±6.1-, and 0.31±0.08-fold, respectively) (Figure 2C), and reduced LDH activity 4.1±0.5% (*p*<0.05). However, unlike norUDCA, UDCA treatment did not significantly alter cell proliferation (Figure 2B). Also, norUDCA treatment showed a 1.3±0.6% better reduction in LDH activity compared to UDCA treatment after injury (*p*<0.05) (Figure 2D). Taken together, these data show that after our standard cytokine + hydrophobic bile acid exposure (TNF-α+TCA), both norUDCA and UDCA treatments reduce cholangiocyte reactive phenotype activation and cytotoxicity. However, norUDCA was more effective than UDCA in reducing cytotoxicity and promoting reparative cholangiocyte proliferation after injury.

FIGURE 2Protective effects of norUDCA and UDCA treatments in primary human cholangiocyte model. Extrahepatic cholangiocyte organoids were exposed to either Veh control or 20 ng/mL TNF-α+1 mM TCA for 8 hours to induce injury, then treated with either Veh or 250 µM NorUDCA or 62.5 µM UDCA for a total of 24 hours. (A) Extrahepatic cholangiocyte organoid diameter measured before and 24 hours after injury indicating protection from diameter loss with both treatments, 20–40 extrahepatic cholangiocyte organoids measured/sample and graphed relative to Veh, images depict 24-hour end point. (B) Immunostaining with cell proliferation marker Ki67 (green) and nuclear marker DAPI (blue) indicating improvement in cell proliferation with norUDCA treatment after injury, proliferation quantified as % Ki67+ cells relative to total cells/field, 6–8 random fields/sample were counted. (C) Gene expression analysis by quantitative real-time PCR demonstrating protection from reactive phenotype marker upregulation with both norUDCA and UDCA treatments after injury. Graphs depict fold change relative to Veh. (D) % LDH activity indicating a greater reduction in cytotoxicity after norUDCA than UDCA treatment after injury, quantified using LDH assay in cell culture media. N = 8 to 12, error bars represent SD, *p*<0.05, *compared to Veh, #compared to TNFα+TCA injury alone, ϕcompared between norUDCA and UDCA. Abbreviations: LDH, lactate dehydrogenase; MCP-1, monocyte chemoattractant protein-1; TCA, taurocholic acid; Veh, vehicle; UDCA, ursodeoxycholic acid; Vim, vimentin.

**Figure:**
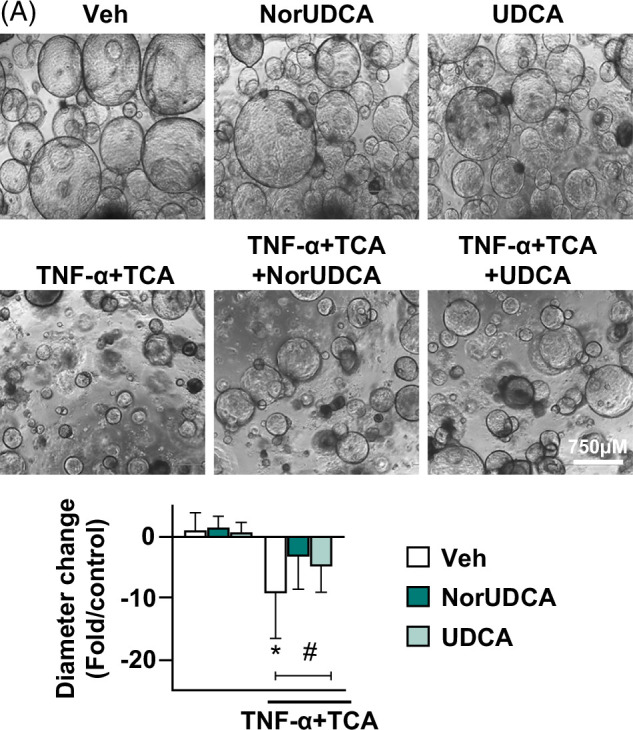

**Figure:**
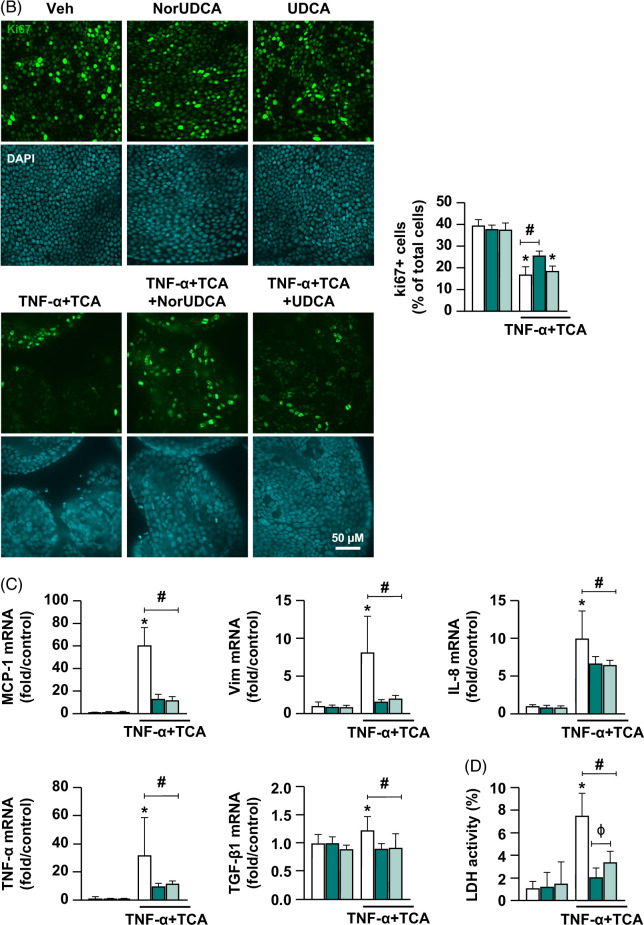

### Role of the S1PR2 pathway in cholangiocyte injury and reactive phenotype activation

**FIGURE 3:**
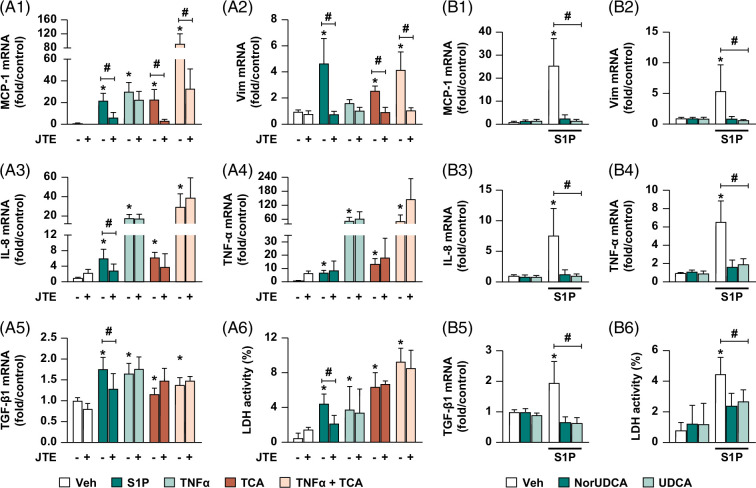
Role of S1PR2 pathway in cholangiocyte injury model and treatment effects. (A) S1PR2 inhibition blocks S1P-induced cholangiocyte-reactive phenotype activation and LDH activity; and partially blocks TCA and TNF-α+TCA–induced reactive phenotype activation. Extrahepatic cholangiocyte organoids were stimulated with either Veh control, 10 µM S1PR2 agonist S1P, 1 mM TCA, 20 ng/mL TNF-α or TNF-α+TCA for 24 hours; in indicated groups, 10 µM S1PR2 antagonist JTE-013 (JTE) was added 30 minutes prior to stimulation to block S1PR2 signaling. (A-1 to 5) Cholangiocyte reactive phenotype marker expression analyzed by quantitative real-time PCR and graphed as fold change relative to Veh. (A-6) % LDH activity in cell culture media quantified using LDH assay. (B) NorUDCA and UDCA treatments suppress S1P-induced reactive phenotype activation and LDH activity. Extrahepatic cholangiocyte organoids were exposed to either Veh or 10 µM S1P for 8 hours, then treated with either Veh, 250 µM norUDCA, or 62.5 µM UDCA for a total of 24 hours. (B-1 to 5) Cholangiocyte-reactive phenotype marker expression analyzed by quantitative real-time PCR and graphed as fold change relative to Veh. (B-6) % LDH activity quantified using LDH assay. N = 4–8, error bars represent SD, *p*<0.05; (A) *compared to Veh, #compared between±JTE within injury group. (B) *compared to Veh, #NorUDCA, or UDCA compared to S1P alone. Abbreviations: JTE-013 sphingosine 1-phosphate receptor 2/4 antagonist; LDH, lactate dehydrogenase; MCP-1, monocyte chemoattractant protein-1; S1P, sphingosine-1-phosphate; TCA, taurocholic acid; UDCA, ursodeoxycholic acid; Veh, vehicle; Vim, vimentin.

**FIGURE 4:**
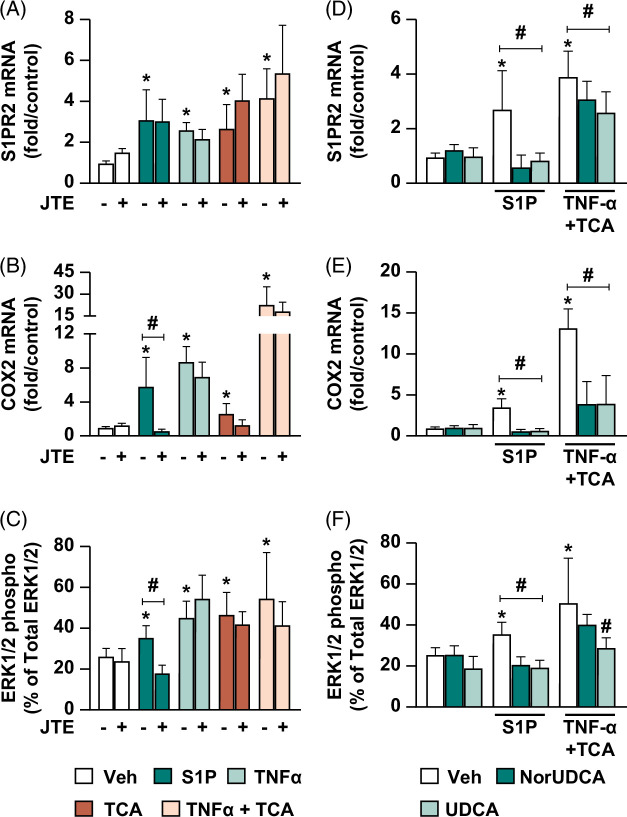
S1PR2 pathway mediators are upregulated in the cholangiocyte injury model and can be modulated by norUDCA or UDCA treatment. (A–C) Cholangiocyte injury by S1P, TNF-α, TCA, or TNF-α+TCA upregulates S1PR2 and its downstream mediators. Extrahepatic cholangiocyte organoids were stimulated with either Veh control, 10 µM S1PR2 agonist S1P, 1 mM TCA, 20 ng/mL TNF-α or TNF-α+TCA for 24 hours; in indicated groups, 10 µM S1PR2 antagonist JTE-013 (JTE) was added 30 minutes prior to stimulation to block S1PR2 signaling. (A) S1PR2 and (B) COX-2 gene expression analyzed by quantitative real-time PCR and graphed as fold change relative to Veh, and (C) % phosphorylated ERK1/2 relative to total ERK1/2 measured by ELISAs. (D–F) NorUDCA and UDCA treatments after S1P or TNF-α + TCA–induced injury suppress S1PR2 and its downstream mediator upregulation. Extrahepatic cholangiocyte organoids exposed to either Veh, 10 µM S1P, or 20 ng/mL TNF-α+1 mM TCA for 8 hours to induce injury, then treated with either Veh, 250 µM norUDCA, or 62.5 µM UDCA for a total of 24 hours. S1PR2 (D) and COX2 (E) gene expression analyzed by quantitative real-time PCR and graphed as fold change relative to Veh; (F) % phosphorylated ERK1/2 relative to total ERK1/2 detected by ELISAs. N = 4–8, error bars represent SD, *p*<0.05, (A–C) *compared to Veh, #compared between±JTE within injury group; (D–F) *compared to Veh, # norUDCA or UDCA compared to S1P or TNFα+TCA injury alone. Abbreviations: COX2, cyclooxygenase-2; ERK1/2, extracellular signal-regulated kinase 1/2; JTE-013, sphingosine 1-phosphate receptor 2/4 antagonist; S1P, sphingosine-1-phosphate; S1PR2, sphingosine-1-phosphate receptor 2; TCA, taurocholic acid; UDCA, ursodeoxycholic acid; Veh, vehicle.

**FIGURE 5:**
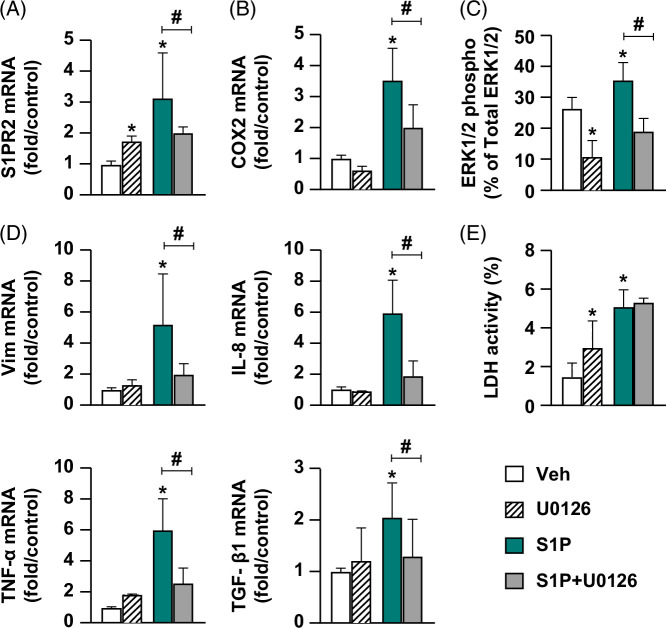
ERK1/2 phosphorylation may be one of the downstream mediators for S1PR2-induced cholangiocyte-reactive phenotype. Extrahepatic cholangiocyte organoids were exposed to either Veh or 10 µM S1P with or without costimulation of ERK phosphorylation inhibitor 2 µM U0126 for 24 hours. (A) S1PR2 and (B) COX2 gene expression analyzed by quantitative real-time PCR relative to Veh; (C) % phosphorylated ERK1/2 relative to total ERK1/2 detected by ELISAs, (D) gene expression of reactive phenotype markers analyzed by quantitative real-time PCR relative to Veh; (E) % LDH activity in cell culture media quantified using LDH assay. Inhibiting ERK1/2 phosphorylation downstream of S1PR2 can suppress S1PR2 pathway mediators, cholangiocyte-reactive phenotype marker upregulation, and LDH activity. N = 4–6, error bars represent SD, *p*<0.05, * compared to Veh, # S1P+U0126 compared to S1P alone. Abbreviations: COX2, cyclooxygenase-2; ERK1/2, extracellular signal-regulated kinase 1/2; LDH, lactate dehydrogenase; S1P, sphingosine-1-phosphate; S1PR2, sphingosine-1-phosphate receptor 2; Vim, vimentin.

**FIGURE 6:**
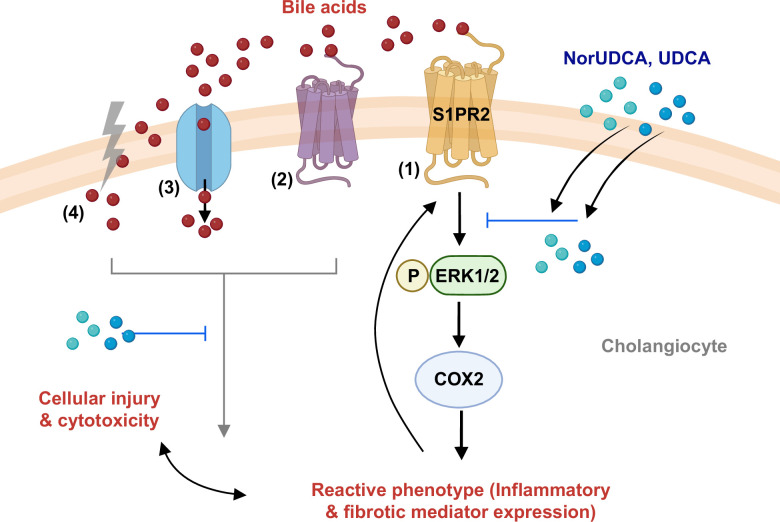
Putative mechanisms of cholangiocyte injury and treatment. (1) Conjugated bile acids can bind and activate the transmembrane receptor S1PR2, leading to signal transduction via ERK1/2 phosphorylation. This promotes upregulation of downstream cell signaling and transcription regulators such as COX-2, which is a known regulator of inflammatory and fibrotic responses in cholangiopathies. The paracrine mediators released from injured cholangiocytes may initiate positive feedback responses on the S1PR2 pathway and can also contribute to cellular injury and cytotoxicity. (2) Signaling through other transmembrane receptors, (3) bile acid uptake via transporters, and (4) passive diffusion of hydrophobic bile acids through the cell membrane may also contribute to cholangiocyte injury and reactive phenotype activation. NorUDCA and UDCA treatments may protect cholangiocytes from injury by suppressing the S1PR2 pathway and also through modulation of the other injury mechanisms involved. Abbreviations: COX2, cyclooxygenase-2; ERK1/2, extracellular signal-regulated kinase 1/2; S1PR2, sphingosine-1-phosphate receptor 2; UDCA, ursodeoxycholic acid.

In order to explore potential roles for S1PR2 signaling on cholangiocyte reactive phenotype in ECOs, we exposed these cells to either the S1PR2-specific agonist S1P (10 µM), TNF-α (20 ng/mL), TCA (1 mM), or a combination of TNF-α+TCA. After exposure to any of these treatments, cholangiocyte reactive phenotype marker expressions were significantly increased compared to vehicle control (Figure 3A-1 to 5). Concomitant treatment of cells with the S1PR2 antagonist JTE-013 (JTE, 10 µM) attenuated S1P induction of the reactive phenotype markers for most of the test genes, specifically MCP-1 and Vim by 15.5±6.4- and 3.9±0.4-fold, respectively, compared to S1P alone (Figure 3A-1, 2). TNF-α induction of test-reactive phenotype genes was poorly reduced by JTE treatment. However, the induction of MCP-1 and Vim by both TCA and TNF-α+TCA was significantly reduced by JTE treatment:19.7±6.9- and 1.6±0.4-fold, respectively, compared to TCA alone; and 59.2±6.3- and 3.1±0.4-fold, respectively, compared to TNF-α+TCA injury alone (*p*<0.05) (Figure 3A-1, 2). Finally, JTE pretreatment attenuated S1P-induced LDH activity by 2.3±0.9% compared to S1P alone (*p*<0.05) (Figure 3A-6) but had no significant effect on the other 3 treatments. Taken together, the S1PR2 inhibitor JTE was able to reduce several aspects of the reactive ductular phenotype of ECOs induced by S1P and bile acids.

Next, we examined whether norUDCA or UDCA treatment can suppress S1P-induced cholangiocyte reactive phenotype activation and LDH activity. When norUDCA or UDCA treatments were given 8 hours after S1P exposure, both treatments significantly suppressed the expression of reactive phenotype markers MCP-1 22.8±2.9- and 24±2.9-fold; Vim 4.5±1.1- and 4.8±1.1-fold; IL-8 6.4±1.1- and 6.6±1.1-fold; TNF-α 5±0.6- and 4.7±0.6-fold; TGF-β1 1.29±0.2- and 1.3±0.2-fold, respectively, compared to S1P alone (*p*<0.05) (Figure 3B-1 to 5). NorUDCA or UDCA treatments also reduced LDH activity by 2.1±0.5 and 1.8±0.5%, respectively, compared to S1P alone (*p*<0.05) (Figure 3B-6). These studies indicate a potential positive anti-inflammatory effect of these 2 bile acids on S1P-mediated pathways.

### Downstream mediators in the S1PR2 pathway and their role in cholangiocyte injury with and without norUDCA or UDCA treatment

S1PR2 and its putative downstream mediators ERK1/2 and COX-2^25^,^38^ were evaluated after exposure to the same treatments as above. Compared to vehicle control, S1P, TNF-α, TCA, or TNF-α+TCA exposure upregulated S1PR2 gene expression 3.1±1.5-, 2.6±0.4-, 2.7±1.1-, and 4.2±1.4-fold, respectively (Figure 4A); upregulated COX-2 gene expression 5.9±3.4-, 8.8±1.7-, 2.7±1.1-, and 23±12-fold, respectively (Figure 4B); and increased ERK1/2 phosphorylation 9.2±5.1, 19±6.4, 20.4±5.5, and 28.3±5%, respectively (Figure 4C) (*p*<0.05). JTE treatment antagonized S1P-induced COX-2 expression by 5.2±2.1-fold (Figure 4B) and ERK1/2 phosphorylation by 17.5±5.9% (Figure 4C) compared to S1P alone (*p* <0.05). Significant changes in these markers were not observed after JTE cotreatment with TNF-α, TCA, or TNF-α+TCA–induced injury. Taken together, these studies indicate ERK1/2 and COX-2 could be downstream mediators of S1PR2 signaling in human cholangiocytes.

Next, we explored whether norUDCA or UDCA treatments can modulate S1PR2 pathway mediators. NorUDCA or UDCA treatments significantly reduced S1P-induced expression of S1PR2 2.1±0.4- and 1.9±0.4-fold, respectively (Figure 4D); reduced COX-2 expression 2.1±0.4- and 1.9±0.4-fold, respectively (Figure 4E); and reduced ERK1/2 phosphorylation 14.8±2.5 and 16.2±2.5%, respectively (Figure 4F) compared to S1P (*p*<0.05). Similarly, compared to TNF-α+TCA, both norUDCA or UDCA treatments reduced S1PR2 expression 0.8±0.3- and 1.3±0.3-fold (Figure 4D) and COX-2 expression 9.2±2.6- and 9.1±0.3-fold (Figure 4E), respectively; UDCA treatment also reduced ERK1/2 phosphorylation 22±8.2% compared to TNF-α+TCA alone (Figure 4F) (*p*<0.05). These experiments suggest that both norUDCA and UDCA treatments can suppress S1PR2 pathway mediators after S1P stimulation and also under cholestatic conditions.

To examine the role of ERK1/2 phosphorylation downstream of S1PR2 activation, we used the ERK1/2 phosphorylation inhibitor U0126 following either vehicle or S1P exposure. Compared to S1P alone, U0126 attenuated S1PR2 expression by 1.14±0.4-fold (Figure 5A); COX-2 expression by 1.5±0.4-fold (Figure 5B); ERK1/2 phosphorylation by 16.5±2.8% (Figure 5C); and reduced mRNA levels of the reactive phenotype markers Vim, IL-8, TNF-α, and TGF-β1 by 3.2±0.9-, 4.1±0.8-, 3.4±0.8-, and 0.8±0.3-fold, respectively (Figure 5D) (*p*<0.05). LDH activity was increased after S1P stimulation compared to Veh; however, U0126 cotreatment did not suppress this effect (Figure 5E). Taken together, these data suggest ERK1/2 as a possible downstream mediator for some of the S1PR2-induced cholangiocyte reactive responses.

## DISCUSSION

The cholangiocyte reactive phenotype is a central pathophysiologic feature in many cholestatic diseases.^14^ In cholestasis, cholangiocytes are exposed to a potentially toxic mixture of high concentrations of hydrophobic bile acids and proinflammatory mediators. How these stimuli induce the cholangiocyte-reactive phenotype and the mechanisms involved are not clearly understood and have not been explored in detail in human cells. Our goal was to create a robust primary human cholangiocyte injury model that emulates key aspects of cholestatic conditions in vitro to study the mechanisms underlying cholangiocyte injury and reactive phenotype activation.

The main findings of this study are (1) hydrophobic primary human bile acids elicit a reactive phenotype activation at high concentrations, many of these injury parameters can be aggravated by proinflammatory cytokine cotreatment; (2) both norUDCA and UDCA treatments are effective in suppressing cholangiocyte injury and reactive phenotype activation in the in vitro cholestatic condition; (3) the S1PR2 pathway may partially contribute to cholangiocyte-reactive phenotype activation after bile acid stimulation, and ERK1/2 and COX-2 signaling could be important downstream effectors; (4) both norUDCA and UDCA appear to protect cholangiocytes from injury and reactive phenotype activation in part due to suppression of the S1PR2 pathway.

Bile acid composition analyses in cholestatic patient serum^16^ and bile^18^ show markedly increased total bile acid levels. In biliary disorders, total ductular bile acid concentrations can range between 5 and 10 mM, which can be attributed predominantly to millimolar concentrations of primary conjugated bile acids (TCA 2–4 mM, GCA 1.5–2.5 mM, GCDCA 1–2.5 mM, and TCDCA 1–2 mM).^18^,^39^ In our model, we tested these pathophysiological millimolar concentrations of three of the most abundant conjugated primary bile acids TCA, GCA, and GCDCA. Data from our injury models reveal that these high concentrations of bile acids can induce cholangiocyte injury and reactive phenotype activation, evident from their loss of diameter and proliferation, increase in membrane permeability and senescence, and upregulation of select reactive phenotype marker mRNAs. The bile acid concentrations needed to induce injury may correlate with their hydrophobicity and the implicit molecular characteristics of each bile acid.^40^ We observed that the more hydrophobic bile acid GCDCA induced injury at lower concentrations than the less hydrophobic bile acids TCA or GCA. As expected, combinations of bile acids and the inflammatory mediator TNF-α as a seminal component of cholestatic conditions further increased injury and reactive phenotype expression. The TNF-α+TCA injury model was selected in this study as TCA is one of the most accumulating bile acids in the cholestatic liver.^41^

UDCA treatment in animal models of cholestasis appears to elicit its beneficial effects mainly by restoration of biliary bicarbonate secretion, improvement of choleresis,^42^,^43^,^44^ and protection from cellular senescence and autophagy.^45^ NorUDCA (a side chain shortened synthetic version of UDCA) has shown promising results in a recent phase 2 clinical trial of primary sclerosing cholangitis.^2^ In some disease models of biliary obstruction and primary sclerosing cholangitis, norUDCA treatment showed enhanced protective effects compared to UDCA,^3^,^4^,^5^ which may be attributed to norUDCA’s ability to undergo cholehepatic shunting and enhance biliary bicarbonate secretion,^7^ immunomodulation,^46^ and promotion of cellular proliferation and tight junction integrity.^21^,^47^ However, very little is known about the molecular mechanistic role of either norUDCA or UDCA on cholangiocyte injury and reactive phenotype activation under cholestatic conditions. In our study, norUDCA was better tolerated at high dosages than UDCA; at high doses, UDCA had detrimental cytotoxic effects. In our TNF-α+TCA injury model, both norUDCA and UDCA treatments ameliorated the loss of cyst diameter associated with injury, suppressed the upregulation of cholangiocyte-reactive phenotype markers, and reduced LDH activity associated with cytotoxic effects of injury. NorUDCA also showed a superior ability to suppress cytotoxicity than UDCA in our model. These findings show that norUDCA and UDCA treatments can ameliorate human cholangiocyte injury and reactive phenotype activation under cholestatic conditions. Interestingly, only norUDCA was able to improve cell proliferation after injury.

Cholangiocyte injury and activation of the reactive phenotype is a complex process that may be regulated via multiple signaling pathways. A recent study of obstructive cholestasis in a mouse model detected S1PR2 upregulation in cholangiocytes after bile duct ligation, and following TCA stimulation of isolated mouse cholangiocytes.^25^ S1PR2 is also upregulated in livers of patients with cholestasis.^26^,^27^ Genetic knockout or inhibition of the S1PR2 receptor with antagonist JTE-013 reduced injury and fibrosis associated with bile duct ligation. This was associated with increased ERK1/2 phosphorylation and upregulation of COX-2, a potent modulator of the cellular stress response downstream of S1PR2.^25^,^38^

To test the relevance of the S1PR2 pathway in human cholangiocyte–reactive phenotype activation, damage, and treatment, we first explored whether the S1PR2 pathway agonist S1P and antagonist JTE can modulate these responses in our standard TNF-α+TCA injury model. When the S1PR2 pathway was specifically activated by S1P, the cholangiocyte-reactive phenotype activation profile was recapitulated together with increased LDH activity; these effects were blocked by the S1PR2 antagonist JTE. This suggests that S1PR2 pathway activation can lead to human cholangiocyte reaction and injury. When the antagonist JTE was applied to the injury model, some of the reactive phenotype markers were suppressed, suggesting partial contribution of the S1PR2 pathway in cholangiocyte-reactive phenotype activation under cholestatic injury conditions. As expected, JTE did not block any of the markers associated with TNF-α exposure alone, as S1PR2 is a receptor for conjugated primary bile acids.^48^ However, both norUDCA and UDCA treatments ameliorated the reactive phenotype and LDH activity associated with S1PR2 pathway activation by S1P. This, together with norUDCA and UDCA’s ability to ameliorate injury and reactive phenotype after TNF-α+TCA exposure, indicates that the protective effects of these treatments likely encompass multiple contributing pathways, and the S1PR2 pathway may be an important component.

Next, we explored the effects of injury and treatments on some of the known downstream mediators of the S1PR2 pathway—ERK1/2 and COX-2.^25^,^38^ Direct activation of S1PR2 by its agonist S1P increased S1PR2 expression, ERK1/2 phosphorylation, and COX-2 expression; these effects were suppressed by antagonist JTE. These observations demonstrate the involvement of the ERK1/2-COX-2 signaling axis downstream of S1PR2 activation in primary human cholangiocytes. Cholestatic injury conditions also upregulated these mediators, but JTE did not show significant suppressive effects, further suggesting the involvement of multiple interconnected pathways that may exert positive feedback to S1PR2. However, when norUDCA or UDCA treatments were administered either after specific S1PR2 activation by S1P or after TNF-α+TCA injury, the S1PR2 downstream mediators were suppressed. This suggests that norUDCA and UDCA may directly modulate the S1PR2 pathway and may also indirectly interact with other adaptive feedback mechanisms involved in cellular injury under cholestatic conditions. Using U0126, an inhibitor for ERK1/2 phosphorylation, we showed that ERK1/2 is indeed a downstream regulator of S1PR2 signaling in human cholangiocytes and that it can directly contribute to cholangiocyte-reactive phenotype activation. Taken together, our observations not only highlight the S1PR2 pathway as an important mediator of cholangiocyte injury and a potential target for treatment but also underline the importance of further mechanistic studies needed to discover other contributing pathways involved. Furthermore, this indicates that both norUDCA and UDCA can modulate S1PR2 signaling in cholangiocytes to suppress reactive phenotype activation and injury. These putative mechanisms of cholangiocyte injury in response to bile acids and the effect of norUDCA and UDCA treatments are depicted in the cartoon diagram in Figure 6.

In summary, by employing a primary human cholangiocyte model that incorporates the toxic microenvironmental conditions of the cholestatic liver in vitro, we were able to explore specific aspects of their molecular and cellular responses. These studies demonstrate that pathological concentrations of bile acids can induce cholangiocyte injury and the reactive phenotype, which can be aggravated by the presence of a proinflammatory mediator. Both norUDCA and UDCA treatments can reduce human cholangiocyte injury and reactive phenotype activation under test cholestatic conditions. On a molecular level, these studies highlight the S1PR2 pathway as one of the drivers of human cholangiocyte injury and reactive phenotype activation and that the S1PR2 pathway may be an important component of norUDCA’s and UDCA’s efficacy. We anticipate that this model can be used as a platform to test other cholestatic injury mechanisms and therapeutic interventions for human cholangiocytes.

## Supplementary Material

SUPPLEMENTARY MATERIAL
