# Supplementary material for: A novel model to study mechanisms of cholestasis in human cholangiocytes reveals a role for the SIPR2 pathway
Source: Hepatol Commun. 2024 Feb 26;8(3):e0389. doi: 10.1097/HC9.0000000000000389 (PMC10898671; doi:10.1097/HC9.0000000000000389)
Supplement: SUPPLEMENTARY MATERIAL [file hc9-8-e0389-s001.docx]

**HEP4-23-0643**

**A Novel Model to Study Mechanisms of Cholestasis in Human Cholangiocytes Reveals a Role for the SIPR2 Pathway**

**Supplementary Methods**

***Propidium Iodide staining***

In 6 experiments ECOs were cultured in glass bottom chamber slides for 5 days (80807, IBIDI) and then exposed to either vehicle (DMSO) or the following bile acids individually for 24 h: 5, 1, 0.5 mM TCA; 5, 1, 0.5 mM GCA; or 1, 0.5, 0.25 mM GCDCA. After 24 h, ECOs were incubated in cell membrane impermeant nuclear stain Propidium Iodide (PI) (Invitrogen, P3566) 10 µg/ml in PBS for 30 min at 37^o^C to stain dead cells, washed 3 times with warm PBS, fixed with 4% Paraformaldehyde (Sigma) at room temperature for 20 min, and counterstained with DAPI (Invitrogen) to label all nuclei. Images were acquired with spinning disc confocal microscope (Leica) at 20X magnification and analyzed in Velocity 7 software (Quorum Technologies Inc.). Percentage of PI positive cells in each image field was calculated as [(# of PI+ nuclei) / (# of DAPI+ nuclei) X 100]. 4 random fields were analyzed/ sample.

***Cleaved caspase 3 immunostaining & Caspase 3 activity assay***

Organoids were plated in 8-well chamber slides (ibidi). After appropriate treatments with vehicle or bile acids ± TNF-α for 24 h the organoids were fixed in 4% formaldehyde (Polysciences Inc.) for 20 min at room temperature, permeabilized with 100 mM Glycine (Multicell/Wisent Inc.) + 0.1% Triton X-100 (BioBasic) for 20 min and then blocked for 1 hr in 5% BSA (Bioshop) + 0.1% Triton X-100. Anti-cleaved caspase-3 primary antibody (9661, Cell Signaling) and AlexaFluor 488 secondary antibody (Invitrogen) were used to detect cleaved caspase 3 positive cells. DAPI (Invitrogen) was used to stain nuclei. 6 to 10 random fields were imaged at 200X magnification using Leica SP8 lightning confocal microscope (Leica, Wetzlar, Germany). Cleaved caspase-3 positive cells were counted using Leica LAS X core software. Total cells were counted using Image J2 software (Fiji). Thresholding was applied to each DAPI image to distinguish cells from the background. An area filter range was applied to exclude debris or low signal cells and to prevent counting multiple cells as one. Cleaved caspase 3 positive cells expressed as percentage of total cells/field.

Caspase 3 activity was measured using colorimetric assay kit (ab39401, Abcam) following manufacturer’s instructions. Briefly, organoids were cultured and treated as described above, total cell lysate was used to measure Caspase 3 activity as chromophore p-nitroaniline (p-NA) release after cleavage from labeled substrate DEVD-pNA by Caspase 3 enzyme activity. The p-NA light emission was measured using microtiter plate reader at 405 nm.

***NorUDCA and UDCA dose optimization***

In 3-6 experiments ECOs were exposed to either vehicle or 1mM TCA + 20 ng/ml TNF-α in William’s E medium; 8 h later treatments of either vehicle (DMSO) or NorUDCA or UDCA were given; at doses ranging between 250-1000 µM NorUDCA or 62.5 to 250 µM UDCA; by replacement of 50% of the media with fresh media contacting the respective treatments; for 24 h.

**Supplementary figure legends:**

**Supplementary Fig. 1: Bile acid dose optimization for injury induction.** ECOs were exposed to either vehicle (DMSO); 5, 1, 0.5 mM TCA; 5, 1, 0.5 mM GCA; or 1, 0.5, 0.25 mM GCDCA for 24 h. **(A)** % of Propidium Iodide positive (PI, Red) dead cells calculated relative to DAPI (Blue) stained total cells/field, indicating dose dependent increase in cell death with increased bile acid concentrations. 4 random images analyzed/sample, white triangles indicate examples of PI positive cells. **(B)** % LDH activity in cell culture media quantified using LDH assay showing dose dependent increase in LDH activity with increased bile acid concentrations. N=6, P<0.05, error bars represent SD, * compared to Veh.

**Supplementary Fig. 2: Apoptosis in injury model.** ECOs were exposed to either vehicle control (Veh); 1mM TCA; 1mM GCA; or 0.5mM GCDCA for 24 h. (A) Immunostaining shows representative images of Cleaved caspase 3 positive cells (Green) and nuclei (Blue) at 200x magnification, (B) graph depicts quantification of cleaved caspase 3 positive cells/total nuclei in 6-10 random fields/sample. (C) Caspase 3 activity assay using total cell lysate. N=6, P<0.05, error bars represent SD, * compared to Veh.

**Supplementary Fig. 3: Optimization of NorUDCA and UDCA dose for treatment.** ECOs were first exposed to either vehicle control (Veh) or 20ng/ml TNF-α + 1 mM TCA 1 mM to induce injury. 8 h later cells were treated with either 0.25 mM, 0.5 mM or 1mM NorUDCA; or either 62.5 µM, 125 µM or 250 µM UDCA; samples collected at 24 h. **(A)** % LDH activity in cell culture media quantified using LDH assay and **(B & C)** reactive phenotype markers IL-8 and Vimentin mRNA expression measured by qRT-PCR, graphed relative to Veh. Indicating 0.25 mM NorUDCA and 62.5 µM UDCA to have optimal protective effects against TNF-α+TCA induced LDH activity and reactive phenotype marker up-regulation. N = 3 to 6, error bars represent SD, P<0.05, * compared to Veh, # compared to TNFα+TCA injury alone.

**Supplementary figures:**

**Supplementary Fig. 1**


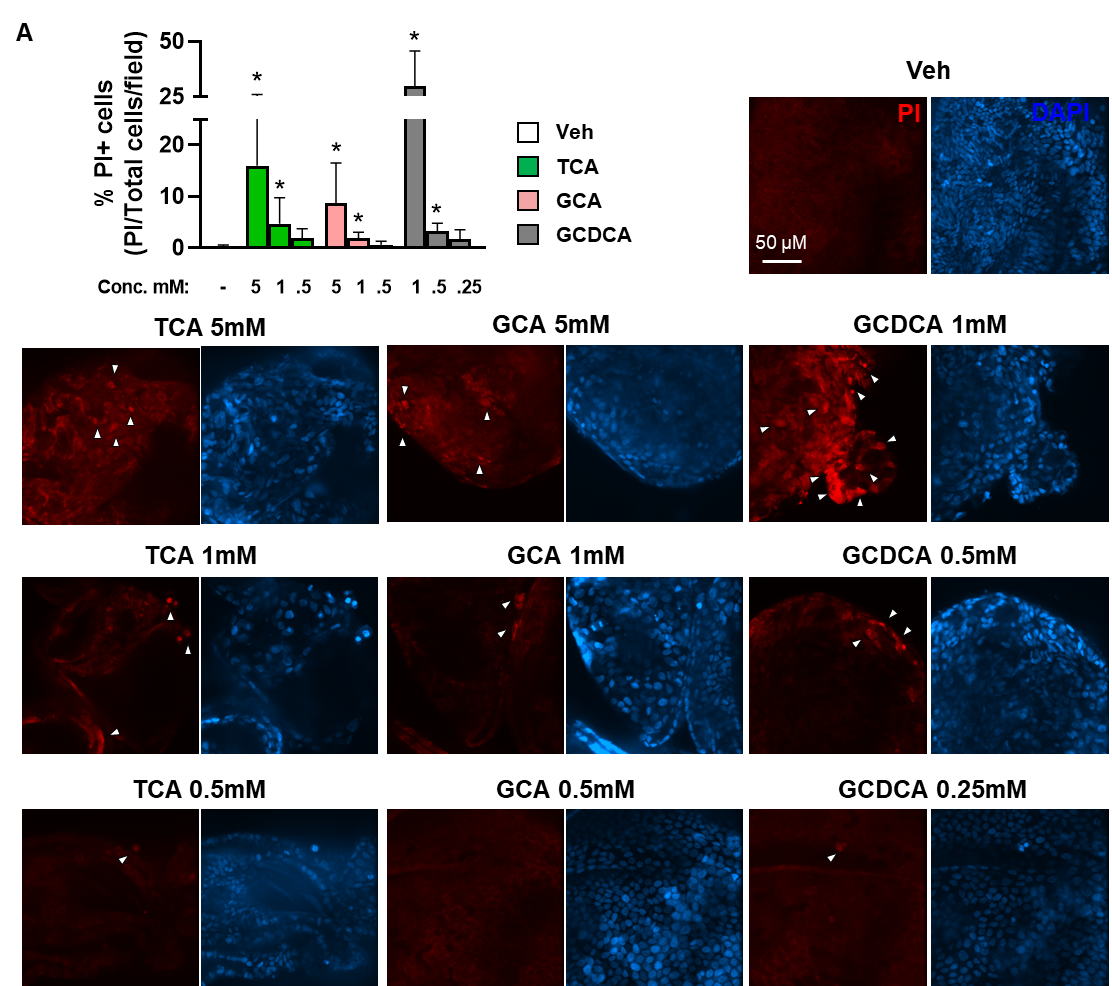


**
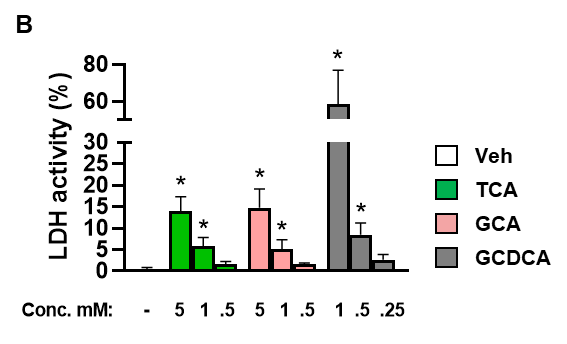
**

**Supplementary Fig. 2**


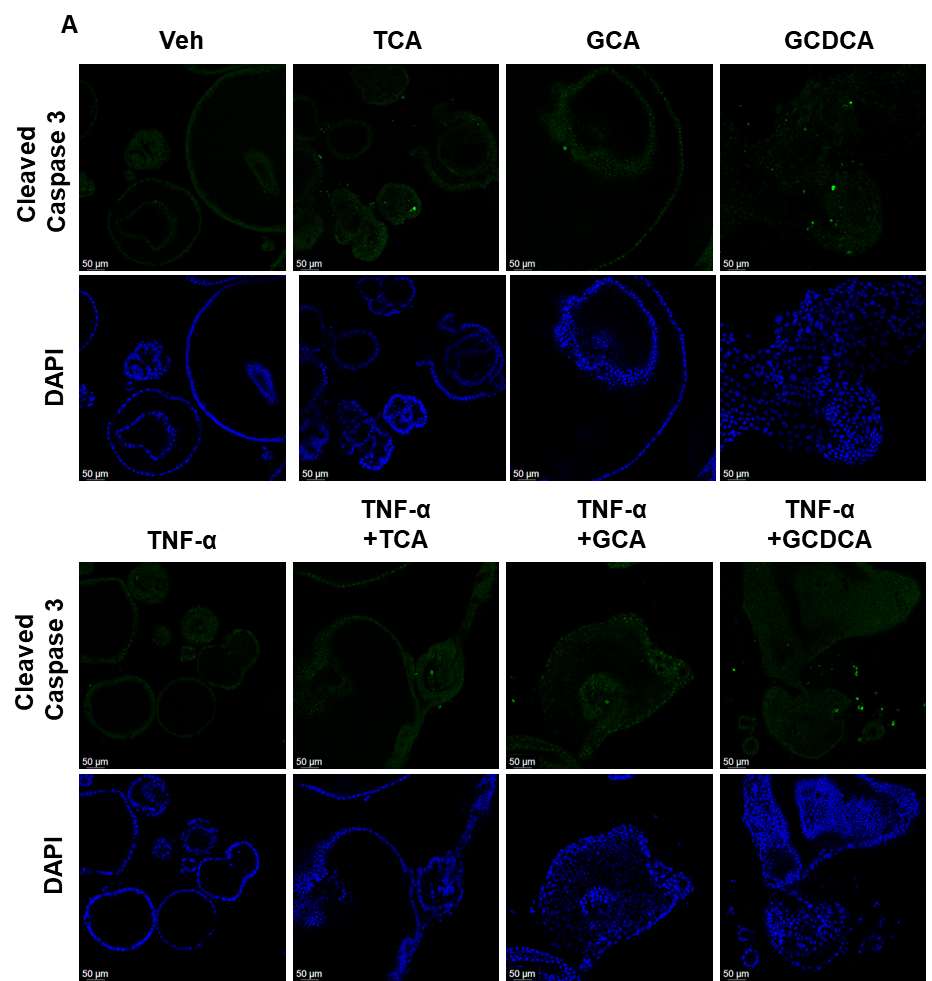


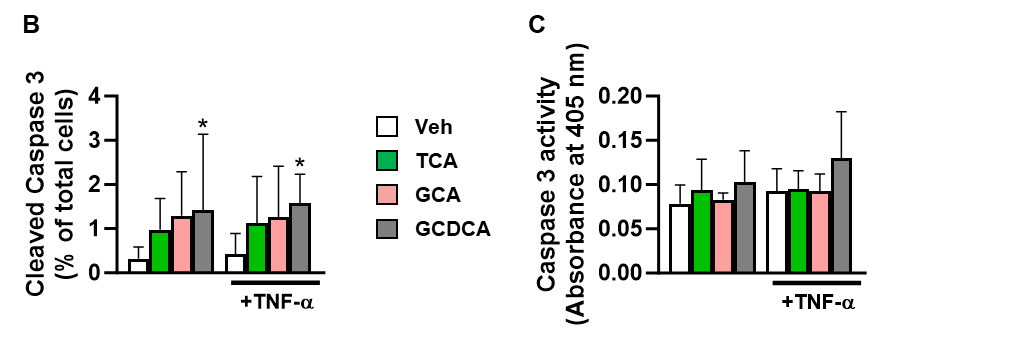


**Supplementary Fig. 3**


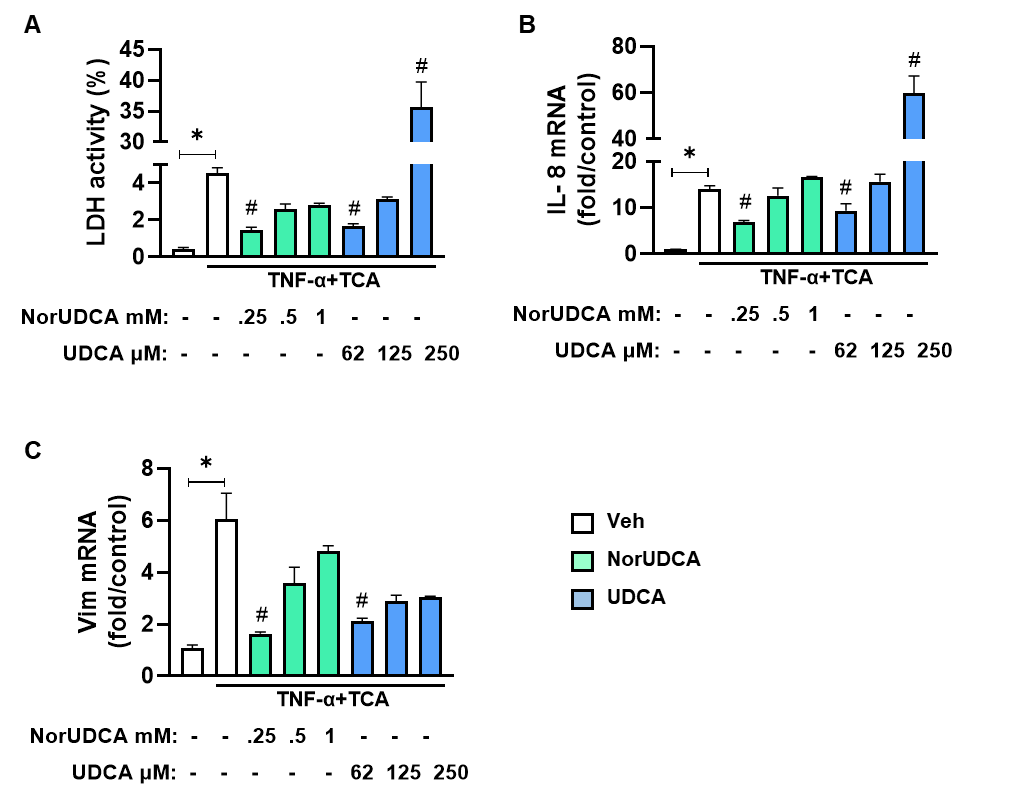


**Supplementary Table 1: Human RT-qPCR Primers**

| **Gene** | **Forward Primer** | **Reverse Primer** |
| --- | --- | --- |
| GAPDH | CTTTGTCAAGCTCATTTCCTGG | TCTTCCTCTTGTGCTCTTGC |
| TNF-α | CCTGCTGCACTTTGGAGTGA | GAGGGTTTGCTACAACATGGG |
| MCP-1 | CCCAAAGAAGCTGTGATCTTCA | TCTGGGGAAAGCTAGGGAA |
| IL-8 | GAAGTTTTTGAAGAGGGCTGAGA | TTTGCTTGAAGTTTCACTGGCA |
| Vimentin | CGGGAGAAATTGCAGGAGGA | AAGGTCAAGACGTGCCAGAG |
| TGF-β1 | GGAAATTGAGGGCTTTCGCC | CCGGTAGTGAACCCGTTGAT |
| S1PR2 | CATCGTCATCCTCTGTTGCG | GCCTGCCAGTAGATCGGAG |
| COX2 | ATGCTGACTATGGCTACAAAAGC | TCGGGCAATCATCAGGCAC |
| p16 | GAGCAGCATGGAGCCTTC | CCTCCGACCGTAACTATTCG |
| p21 | GGCTCCTTCCCATCGCTGTCA | GTCACCCTGCCCAACCTTAGA |
